# Cardamonin Inhibits Oxazolone-Induced Atopic Dermatitis by the Induction of NRF2 and the Inhibition of Th2 Cytokine Production

**DOI:** 10.3390/antiox9090834

**Published:** 2020-09-07

**Authors:** Ok-Kyung Yoo, Won Jun Choi, Young-Sam Keum

**Affiliations:** College of Pharmacy and Integrated Research Institute for Drug Development, Dongguk University, 32 Dongguk-ro, Goyang, Gyeonggi-do 10326, Korea; aamho@naver.com (O.-K.Y.); mp89@dongguk.edu (W.J.C.)

**Keywords:** cardamonin, oxazolone, NF-E2-related factor 2 (NRF2), T helper 2 (Th2) cytokines

## Abstract

The skin is constantly exposed to various types of chemical stresses that challenge the immune cells, leading to the activation of T cell-mediated hypersensitivity reactions including atopic dermatitis. Previous studies have demonstrated that a variety of natural compounds are effective against development of atopic dermatitis by modulating immune responses. Cardamonin is a natural compound abundantly found in cardamom spices and many other medicinal plant species. In the present study, we attempted to examine whether cardamonin could inhibit oxazolone-induced atopic dermatitis in vivo. Our results show that topical application of cardamonin onto the ear of mice suppressed oxazolone-induced inflammation in the ear and hyperplasia in the spleen. Cardamonin also inhibited oxazolone-induced destruction of connective tissues and subsequent infiltration of mast cells into the skin. In addition, we found that the production of Th2 cytokines is negatively regulated by NRF2, and the induction of NRF2 by cardamonin contributed to suppressing oxazolone-induced Th2 cytokine production and oxidative damages in vivo. Together, our results demonstrate that cardamonin is a promising natural compound, which might be effective for treatment of atopic dermatitis.

## 1. Introduction

Atopic dermatitis, also known as atopic eczema, is an inflammatory skin disorder that is characterized by intense pruritus, scratching, and cutaneous hypersensitivity to allergens [1]. It is well established that genetic predisposition is a strong risk factor for the development of atopic dermatitis. Therefore, patients with atopic dermatitis often carry a personal or familial history of other allergic diseases such as asthma and allergic rhinitis, and many of them possess the genetic mutations in the genes associated with defective epidermal differentiation and skin barrier formation [2]. In addition, preferential T helper 2 (Th2) cell-mediated pathway activation [3], neuroimmune interactions [4], and microbial pathogens [5] trigger the development of atopic dermatitis. A hallmark of atopic dermatitis is a dry, itchy, and cracked skin caused by defects in the barrier function of keratinocytes, and current treatment for atopic dermatitis includes topical application of moisturizers, anti-inflammatory agents, and phototherapy [6]. A number of experimental mouse models recapitulating atopic dermatitis have been developed and they are largely categorized into three groups [7]: (i) mouse models that develop atopic dermatitis by cutaneous application of sensitizers, (ii) genetically modified mice that overexpress or lack selective molecules, and (iii) mouse models that spontaneously develop atopic dermatitis-like skin lesions.

Oxidative stress is defined as the formation of oxidants that exceeds the antioxidant defense capacity in cells [8]. It is known that oxidative stress promotes inflammation in the skin by upregulating the pro-inflammatory genes, contributing to the pathogenesis of atopic dermatitis [9]. NF-E2-related factor 2 (NRF2) is a redox-sensitive transcription factor, which is responsible for the induction of phase II cytoprotective enzymes and the detoxification of reactive oxygen species (ROS) by binding to the antioxidant response element (ARE), a *cis*-DNA element existing in the promoter of phase II cytoprotective enzymes [10]. Under basal condition, NRF2 is constantly polyubiqutinated in the cytosol by Kelch-like ECH-associated protein 1 (KEAP1), an adaptor for Cullin 3 (CUL3) E3 ubiquitin ligase. Exposure of oxidants and electrophiles inactivates KEAP1, allows NRF2 to translocate into the nucleus, and activates the expression of NRF2 target genes. Previous studies have demonstrated that NRF2 activators are useful for treatment of various oxidative stress-related diseases, and a number of NRF2 chemical activators are currently undergoing clinical trials for treatment of multiple sclerosis, cardiovascular disease, diabetes mellitus, and psoriasis [11]. However, whether and, if so, how NRF2 activation could effective against atopic dermatitis is still elusive.

**Figure 1:**
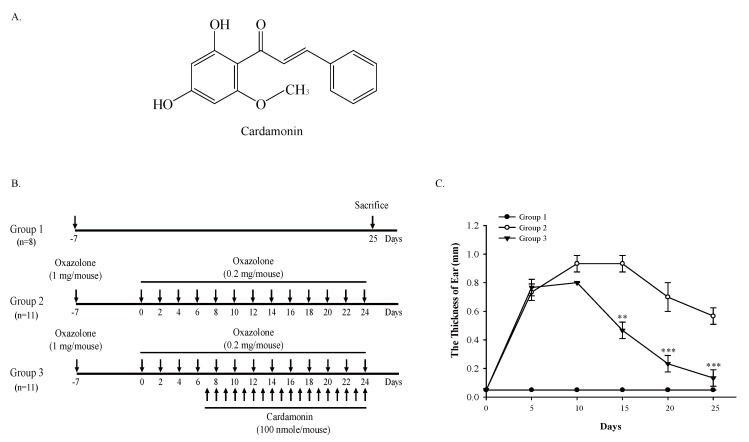
Cardamonin suppresses oxazolone-induced inflammation in mouse skin. (**A**) Chemical structure of cardamonin. (**B**) Experimental scheme evaluating the effect of cardamonin on oxazolone-induced atopic dermatitis. (**C**) Cardamonin inhibits oxazolone-induced inflammation in mouse skin. The thickness of ear in mice was measured using a caliper every five days. Asterisks indicate a statistical significance of ** *p* < 0.01 and *** *p* < 0.001.

Polyphenols are one of the most abundant ingredients found in our diets and chalcones represent an important group in the polyphenolic family compounds [12]. Cardamonin (Figure 1A) is a naturally-occurring compound with a hydroxylated chalcone structure. The production of cardamonin in plants starts with the deamination of phenylalanine into cinnamic acid, which undergoes additional transformation into hydroxylated chalcone via condensation with malonyl-CoA [13]. Previous studies have demonstrated that cardamonin possesses a number of beneficial pharmacological effects, such as anti-inflammatory, anti-neoplastic, vasodilative and anti-infectious effects [14]. In the present study, we have identified that cardamonin inhibits oxazolone-induced atopic dermatitis in vivo by inhibiting the production of Th2 cytokines and subsequent oxidative damages through the induction of NRF2.

## 2. Materials and Methods

### 2.1. Cell Culture, Chemicals, and Reagents

Oxazolone was purchased from Sigma-Aldrich (St. Louis, MO, USA). Cardamonin was purchased from TOCRIS bioscience (Bristol, UK). Dulbecco’s modified Eagle’s medium (DMEM), heat-inactivated fetal bovine serum (FBS), phosphate-buffered saline (PBS), and penicillin/streptomycin (Pen/Strep) were purchased from Welgene (Daegu, Korea). Polyclonal antibodies against NRF2 were purchased from Cell Signaling Technology (Danvers, MA, USA). Monoclonal antibodies against total actin and 8-hydroxydeoxyguanosine (8-OH-dG) were purchased from Santa Cruz Biotechnology (Santa Cruz, CA, USA). Polyclonal antibodies against 4-hydroxynonenal (4-HNE) were purchased from Abcam (Cambridge, MA, USA). Human keratinocyte HaCaT cells were acquired from American Type Culture Collection (Manassas, VA, USA).

### 2.2. Examination of the Effect of Cardamonin on Oxazolone-Induced Atopic Dermatitis

The animal experiment was carried under the Institutional Animal Care and Use Committee-approved Protocol (IACUC-2019-002-1) of Dongguk University (Seoul, Korea). Six-week-old Balb/c mice were purchased from Daehan Biolink (Eumseong, Korea), housed in sterile filter-capped microisolator cages, and provided with water and diet ad libitum. After a week of acclimation, 30 mice were distributed to control (*n* = 8, Group 1), oxazolone (*n* = 11, Group 2), and oxazolone + cardamonin (*n* = 11, Group 3) groups (Figure 1B). After distribution, mice were topically applied with oxazolone alone or in combination with cardamonin on to the ear, and the thickness of the ear was measured by a caliper during the course of experiment. At sacrifice, tissues were excised, weighed, and stored in a deep freezer for biochemical analysis or in 10% formalin solution for immunohistochemistry.

### 2.3. Tissue Dehydration and Paraffin Embedding

At sacrifice, the mouse ears were fixed in 10% formalin solution overnight. Tissue dehydration was performed by serially immersing the tissues into 75%, 80%, 85%, 90%, 95%, and 100% ethanol and xylene solution for 1 h at each step. Dehydrated tissues were embedded in the paraffin block.

### 2.4. Hematoxylin & Eosin (H&E) Staining

Paraffin-embedded tissues were sectioned at 5 μm, mounted on the slide, and deparaffinized. Tissues were stained with Mayer’s hematoxylin solution for 5 min at room temperature, then rinsed in tap water until the water becomes clear. In the bluing step, the tissues were stained with repeated cycles of eosin Y ethanol solution for 70 sec, 5 dips in 95% ethanol, and 5 dips in 100% ethanol at room temperature. The tissues were rinsed with distilled water and the images were taken on the microscope (Olympus, Tokyo, Japan).

### 2.5. Preparation of Primary Mouse Embryonic Fibroblasts (MEFs)

Nrf2 (−/−) mice were purchased from the Jackson Laboratory (Bar Harbor, ME, USA) and bred in the animal facility of Dongguk University. Primary Nrf2 (+/+) and Nrf2 (−/−) mouse embryonic fibroblasts (MEFs) were generated from E12.5 embryos and the genotype was confirmed by PCR analysis. Primary MEFs separated from embryos were cultured in DMEM media containing 10% heat-inactivated FBS and 1× Pen/Strep at 37 °C in humidified 5% CO_2_ incubator.

### 2.6. Masson’s Trichrome Staining

Paraffin-embedded tissues were sectioned at 5 μm, mounted on the slide, and deparaffinized. Tissues were stained with Masson’s trichrome reagents as recommended by the manufacturer (Labcore, Seoul, Korea) and the images were taken on the microscope (Olympus, Tokyo, Japan).

### 2.7. Immunohistochemistry Staining with DAB

Tissues on the slide were incubated with 1% bovine serum albumin (BSA) blocking solution for 30 min. After washing three times with 1× PBS, the tissues were hybridized with primary antibodies overnight at 4 °C. The slides were washed with 1× PBS three times and incubated with anti-rabbit and anti-mouse UltraTEk HRP antibodies (ScyTek Inc., Logan, UT, USA). Development of the slides was performed with 3,3′-diaminobenzidine (DAB) (GBI Labs, Bothell, WA, USA). The slides were then sealed with mounting medium and the images were taken on the microscope (Olympus, Tokyo, Japan).

### 2.8. Measurement of ARE-Luciferase Activity in HaCaT-ARE-Luciferase Cells

Establishment of human keratinocyte HaCaT-ARE-GFP-luciferase cells was previously described [15]. Established HaCaT-ARE-luciferase cells were seeded in six-well plates, cultured until 70% confluence, and exposed to cardamonin. Sulforaphane was used as a positive control. After 24 h, cells were lysed with a luciferase lysis buffer (0.1 M potassium phosphate buffer at pH 7.8, 1% Triton X-100, 1 mM DTT, 2 mM EDTA) and the resulting luciferase activity was measured by the GLOMAX Multi-system (Promega, Madison, WI, USA). The data is depicted as a fold ratio of the firefly luciferase activity, compared with the control after normalization with protein concentration.

### 2.9. Western Blot Analysis

Tissues were ground by pestle and incubated with 200 μL RIPA buffer (50 mM Tris-HCl at pH 8.0, 150 mM NaCl, 1% NP-40, 0.5% sodium deoxycholate, protease inhibitors cocktail) for 1 h on ice. Lysates were collected by centrifugation and protein concentration was measured by BCA Protein Assay Kit (Thermo Fisher, Pittsburgh, PA, USA). Equal amounts of lysates were resolved by SDS-PAGE and transferred to PVDF membrane. The membrane was incubated in blocking buffer (5% skim milk in 1× PBS-0.1% Tween-20, PBST) for 1 h and hybridized with the appropriate primary antibodies in 1× PBS containing 3% BSA overnight at 4 °C. After washing three times with 1× PBST for 30 min, the membrane was hybridized with appropriate HRP-conjugated secondary antibody (Cell Signaling Technology, Danvers, MA, USA) for 1 h at room temperature and washed three times with 1× PBST solution for 30 min. The membrane was visualized using an enhanced chemiluminescence (ECL) detection system. A β-actin blot was used as the control for equal loading of samples.

### 2.10. Real-Time RT-PCR Assay

Total RNA was isolated using a Hybrid-R RNA extraction kit (GeneAll, Seoul, Korea). Total RNA (1 μg) was subject to cDNA synthesis, using PrimeScript RT-PCR kit (TaKaRa Korea, Seoul, Korea). Real-time PCR was performed on a CFX96 instrument (Bio-Rad, Hercules, CA, USA) using EvaGreen Supermix (Bio-Rad, Hercules, CA, USA). The primer sequences used for the quantitation of target genes are illustrated in Table 1. GAPDH was used as an internal control.

### 2.11. Statistical Analysis

Statistical analysis was conducted using Student’s t-tests. Asterisks indicate a statistical significance of * *p* < 0.05, ** *p* < 0.01, and *** *p* < 0.001.

## 3. Results

### 3.1. Cardamonin Suppresses Oxazolone-Induced Atopic Dermatitis In Vivo

Contact hypersensitivity induced by topical application of haptens is a commonly used animal model to study dermal inflammatory responses [16]. Haptens are low molecular weight chemicals that can penetrate into the skin and associate with endogenous proteins, resulting in the generation of immunogenic hapten–protein complexes [17]. Formation of the hapten–protein complex induces the migration of activated Langerhans cells and dermal dendritic cells, where the hapten–protein complex is presented to naïve T cells that generate hapten-specific and skin-homing CD8+ and CD4+ T cells, thereby inducing strong local inflammatory responses [18]. Oxazolone is a hapten experimentally used to induce atopic dermatitis and intestinal bowel disease in vivo, and we used oxazolone to evaluate the inhibitory effects of cardamonin on the development of atopic dermatitis.

**Figure 2:**
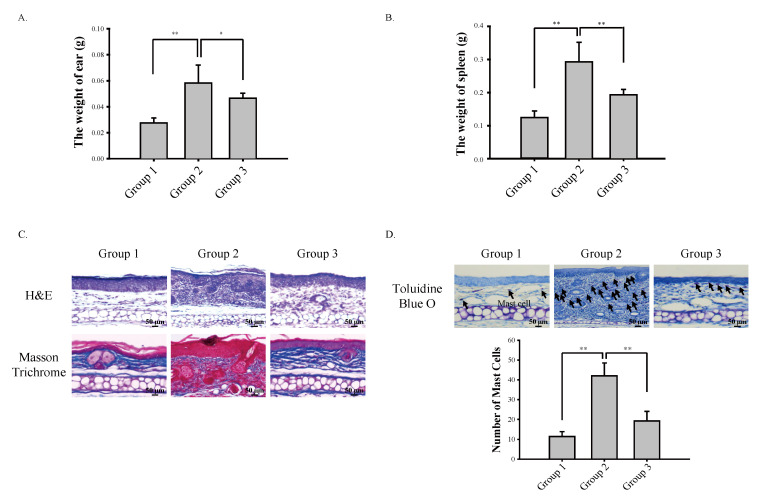
Cardamonin suppresses oxazolone-induced atopic dermatitis by maintaining the integrity of the skin and inhibiting the infiltration of mast cells. (**A**) Cardamonin inhibits an increase in the weight of ear induced by oxazolone. At sacrifice, mouse ears were excised and their weight was measured. Asterisks indicate a statistical significance of * *p* < 0.05 and ** *p* < 0.01. (**B**) Topical application of oxazolone onto the ear of mouse induces splenomegaly in mice, which is inhibited by cardamonin. At sacrifice, the spleen of mouse was excised and the weight was measured. Asterisks indicate a statistical significance of ** *p* < 0.01. (**C**) Cardamonin inhibits oxazolone-induced destruction of connective tissues in the ear of mouse. Gross morphology of mouse skin tissues in the ear are stained by H&E staining (upper panel) and the collagens in connective tissues are stained by Masson trichrome staining (lower panel). (**D**) Cardamonin inhibits oxazolone-induced infiltration of mast cells into the skin. Mast cells are stained by Toluidine Blue O staining (Upper Panel) and the number of mast cells under the bright field was counted (Lower Panel). Asterisks indicate a statistical significance of ** *p* < 0.01.

In the present study, we topically administered oxazolone onto the ear of mice alone or in combination with cardamonin (Figure 1B). During the course of the study, we observed that cardamonin significantly suppressed oxazolone-induced inflammation as measured by the thickness of the ear (Figure 1C). At sacrifice, we observed that cardamonin significantly suppressed oxazolone-induced increase in the weight of the ear (Figure 2A) and splenomegaly (Figure 2B). Immunohistochemistry results showed that cardamonin suppressed oxazolone-induced destruction of connective tissues assessed by Masson trichrome staining (Figure 2C) and subsequent infiltration of mast cells into the epidermis assessed by toluidine blue O staining (Figure 2D). Together, these results suggest that the inhibition of oxazolone-induced atopic dermatitis by cardamonin is associated with suppression of the systemic immune responses.

### 3.2. Cardamonin Suppresses Oxazolone-Induced Production of Th2 Cytokines In Vivo

The immune system is composed of two subsystems: the innate immune system and the adaptive immune system [19]. The innate immune system stands at the frontline of protection against invading pathogens and immediately reacts to infection or trauma. Innate immune cells are equipped with various pattern recognition receptors (PRRs) such as Toll-like receptors (TLRs), nucleotide binding oligomerization domain-like receptors (NLRs), and retinoic acid-inducible gene I-like receptors (RLLs), and they recognize both pathogen-associated molecular patterns (PAMPs) and endogenous damage-associated molecular patterns (DAMPs) [20]. The interaction of PRRs in innate immune cells with PAMPs and/or DAMPs induces the activation of a panel of downstream intracellular signaling pathways including TLR- and NLR-dependent signaling pathways, intracellular kinases, and transcription factors [21]. On the other hand, the adaptive immune system engages the pathogens with specificity and memory. Components of the adaptive immune system include fundamental cells and molecules of the innate immunity such as B lymphocytes (B cells), T lymphocytes (T cells), immunoglobulins, and the major histocompatibility complex (MHC) [22].

**Figure 3:**
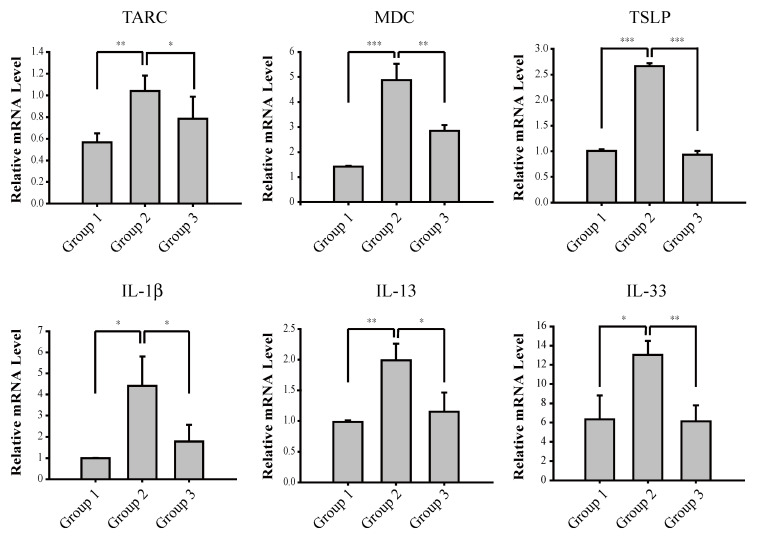
Cardamonin mediates transcriptional suppression of oxazolone-induced production of Th2 cytokines in vivo. The mRNA levels of Th2 cytokines in mouse skin were measured by real-time RT-PCR analysis. Asterisks indicate a statistical significance of * *p* < 0.05, ** *p* < 0.01 and *** *p* < 0.001.

T cells possess the T cell receptor (TCR), which recognizes a specific antigen and is formed using RAG-mediated V[D]J rearrangement. TCR is bound to the membrane and recognize antigens only when presented in the context of MHC I or MHC II [23]. T cells are classified into two main populations: CD8+ T cytotoxic cells that interact with the MHC class I and CD4+ T helper cells that interact with the MHC Class II [24]. T helper cells are an important part of the adaptive immune system [25]. Disruption of epidermal homeostasis polarizes dendritic cells and stimulates Th2 cells to express Th2 cytokines in the skin, contributing to the initiation and progression of atopic dermatitis [26]. Therefore, we next examined whether cardamonin suppresses oxazolone-induced production of Th2 cytokines. Our results show that cardamonin significantly suppressed oxazolone-induced transcription of Th2 cytokines such as thymus- and activation-regulated chemokine (TARC), macrophage-derived chemokine (MDC), thymic stromal lymphopoietin (TSLP), interleukin-1β (IL-1β), interleukin-13 (IL-13), and interleukin-33 (IL-33) in vivo (Figure 3). These results demonstrate that cardamonin suppresses oxazolone-induced Th2 immune responses.

### 3.3. NRF2 Is Responsible for Suppressing Oxazolone-Induced Production of Th2 Cytokines

**Figure 4:**
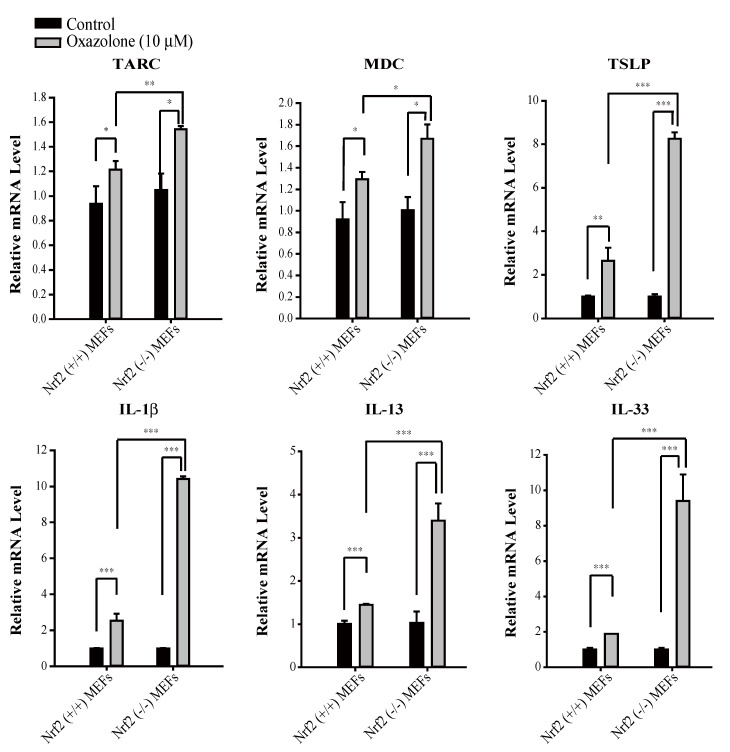
Transcriptional activation of Th2 cytokines by oxazolone is significantly higher in Nrf2 (−/−) MEFs compared with Nrf2 (+/+) MEFs. Primary Nrf2 (+/+) and Nrf2 (−/−) MEFs were exposed to oxazolone (10 μM) for 24 h and real-time PCR was performed using specific primers against Th2 cytokines. Asterisks indicate a statistical significance of * *p* < 0.05, ** *p* < 0.01 and *** *p* < 0.001.

As mentioned earlier, NRF2 is responsible for transcriptional activation of phase II cytoprotective genes. On the other hand, there is also evidence that NRF2 can suppress the production of pro-inflammatory cytokines in certain circumstances [27]. However, the detailed mechanisms underlying how NRF2 suppresses the expression of pro-inflammatory cytokine genes are still unclear. To examine whether NRF2 is implicated in controlling the induction of Th2 cytokines by oxazolone, we prepared primary mouse embryonic fibroblasts (MEFs) from Nrf2 (+/+) and Nrf2 (−/−) mice. Primary Nrf2 (+/+) and Nrf2 (−/−) MEFs were exposed to oxazolone, and the mRNA levels of various Th2 cytokines were assessed by real-time RT-PCR. Our results show that basal level of Th2 cytokines was comparable between Nrf2 (+/+) MEFs and Nrf2 (−/−) MEFs, and that oxazolone caused a significant transcriptional activation of Th2 cytokines in Nrf2 (+/+) MEFs and Nrf2 (−/−) MEFs (Figure 4). Interestingly, we observed that transcriptional activation of Th2 cytokines by oxazolone was significantly higher in Nrf2 (−/−) MEFs, when compared with Nrf2 (+/+) MEFs (Figure 4). This observation suggests that NRF2 exhibits suppressive effects on transcriptional activation of Th2 cytokines by oxazolone.

### 3.4. Cardamonin Induces NRF2 and Attenuates Oxazolone-Induced Oxidative Damages In Vivo

**Figure 5:**
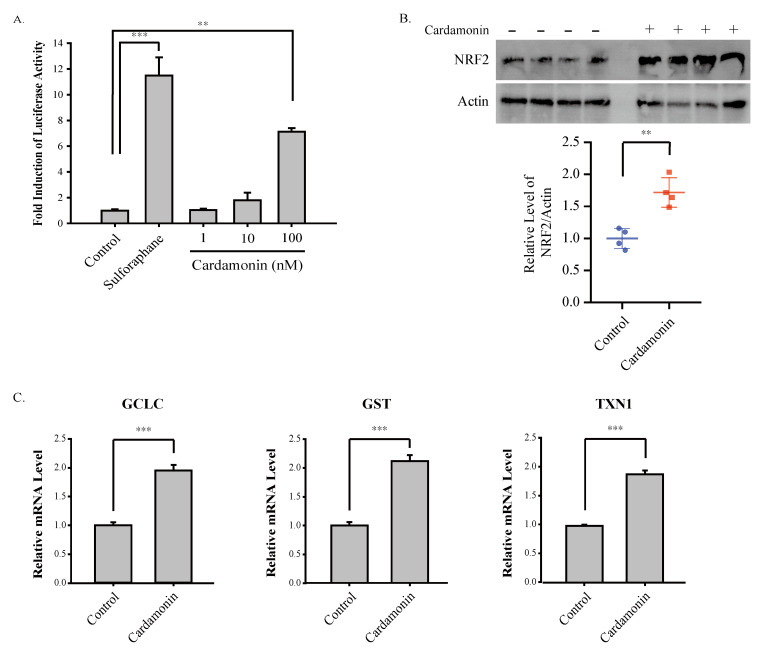
Cardamonin activates ARE-luciferase activity in HaCaT cells and induces the expression NRF2 and its target genes in mice. (**A**) Cardamonin induces ARE activation in HaCaT-ARE-GFP-luciferase cells. HaCaT-ARE-GFP-luciferase cells were exposed to cardamonin at various concentrations for 24 h and the luciferase activity was measured. The experiment was performed in triplicate and sulforaphane (10 μM) was included as a positive control. Asterisks indicate a statistical significance of ** *p* < 0.01 and *** *p* < 0.001. (**B**) Cardamonin induces NRF2 in vivo. Cardamonin (100 nmole/mouse) was topically applied to the ear of mouse for 24 h and Western blotting was performed against NRF2 and actin (Upper Panel). The relative level of NRF2 was normalized against actin (Lower Panel). Asterisks indicate a statistical significance of ** *p* < 0.01. (**C**) Cardamonin induces NRF2 target genes in vivo. Cardamonin (100 nmole/mouse) was topically applied to the ear of mouse for 24 h and real-time RT-PCR was performed against NRF2 target genes (GCLC, GST, TXN1). The experiment was performed in quadruplicate. Asterisks indicate a statistical significance of *** *p* < 0.001.

**Figure 6:**
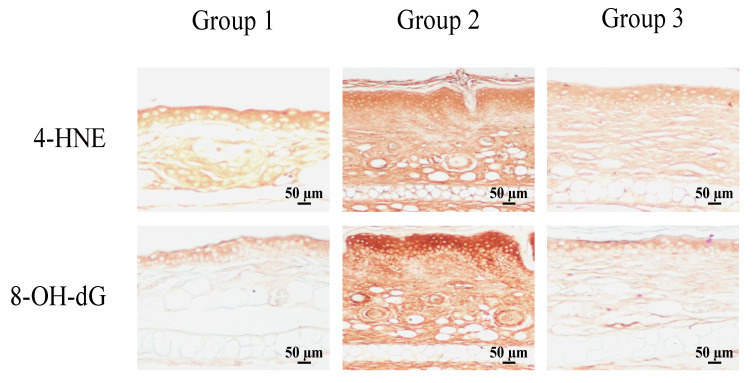
Cardamonin protects against oxazolone-induced oxidative damage in vivo. The formation of oxidative damage markers in mouse skin was visualized by immunohistochemistry using 4-HNE and 8-OH-dG antibodies. A representative slide is provided.

Because NRF2 suppresses oxazolone-induced production of Th2 cytokines (Figure 4), we speculated that cardamonin might be able to activate NRF2, thereby suppressing oxazolone-induced production of Th2 cytokines. Consistent with this notion, cardamonin significantly activated ARE-dependent luciferase activity in HaCaT-ARE-GFP-luciferase cells (Figure 5A). Topical administration of cardamonin onto the ear of mice also induced NRF2 (Figure 5B) and caused a significant transcriptional activation of NRF2 target genes such as glutamate cysteine ligase catalytic subunit (GCLC), glutathione S-transferase (GST), and thioredoxin 1 (TXN1) in vivo (Figure 5C). Finally, we observed that cardamonin suppressed oxazolone-induced oxidative DNA damages in the ear of mice, as revealed by oxidative damage markers such as 4-hydroxynonenal (4-HNE) and 8-hydroxydeoxyguanosine (8-OH-dG) (Figure 6). Together, our results demonstrate that the induction of NRF2 by cardamonin contributes to the inhibition of oxazolone-induced oxidative damages.

## 4. Discussion

We have demonstrated that cardamonin suppresses oxazolone-induced atopic dermatitis in vivo (Figure 1). Suppression of oxazolone-induced atopic dermatitis by cardamonin was associated with maintaining the integrity of the connective tissues (Figure 2C) and inhibiting the production of Th2 cytokines in the skin (Figure 3). We also provided evidence that NRF2 plays a significant role in suppressing oxazolone-induced production of Th2 cytokines (Figure 3). Because production of various Th2 cytokines stimulates B cell proliferation, immunoglobulin class-switching to immunoglobulin E (IgE), and macrophage polarization to an M2-like phenotype [28], suppressing the production of Th2 cytokines by NRF2 could explain, at least in part, how NRF2 modulates the immune system to exert anti-inflammatory responses (Figure 1C). However, the detailed molecular mechanisms underlying how NRF2 affects Th2 cells to attenuate oxazolone-induced production of Th2 cytokines are largely unclear. In addition, we observed that topical administration of oxazolone onto the ear of mice caused splenomegaly (Figure 2B). Considering that Nrf2 (−/−) mice phenotypically exhibit hypertrophy in the spleen [29], it is possible to assume that NRF2 in the spleen might be implicated in allergic responses to oxazolone in keratinocytes. This hypothesis harmonizes with the observation that oxazolone caused a significant accumulation of mast cells in mouse skin (Figure 2D).

Cardamonin stimulates ARE-dependent gene expression (Figure 5A) in HaCaT-ARE-GFP-luciferase cells, and significantly induces the expression of NRF2 (Figure 5B) and its target genes (Figure 5C) in mouse skin. However, the molecular mechanisms underlying the induction of NRF2 by cardamonin remain elusive. One possibility is that the activation of the intracellular kinase pathways by cardamonin such as mitogen-activated protein kinases (MAPK) and phosphatidylinositol 3′-kinase (PI3K) might be responsible for the activation or induction of NRF2 [30]. Alternatively, it can be assumed that the induction of NRF2 by cardamonin occurs via the blockade of poly-ubiquitination of NRF2. Indeed, many NRF2 inducers are known to be Michael acceptors that form direct adducts with cysteine residues in KEAP1 [31]. In view of the structure, cardamonin can be classified as a Michael acceptor because it possesses the α,β-unsaturated lactone moiety (Figure 1A). In addition, the role of E3 ubiquitin ligases other than CUL3/KEAP1 on the induction of NRF2 by cardamonin also merits further exploration, considering that there are multiple E3 ubiquitin ligases that target NRF2 for proteolysis [32].

## 5. Conclusions

In conclusion, our study provides evidence that cardamonin inhibits atopic dermatitis in vivo and that this effect could be attributed to the induction of NRF2 and the inhibition of Th2 cytokine production.

## Figures and Tables

**Table 1:** Sequence of real-time PCR primers.

Gene	Accession Number		Primer Sequence
TARC	NM_011332	Forward Reverse	5′-CTG CTC GAG CCA CCA ATG TA-3′ 5′-TGC CCT GGA CAG TCA GAA AC-3′
MDC	NM_009137	Forward Reverse	5′-GCT GTG GCA ATT CAG ACC TC-3′ 5′-TGA CGG ATG TAG TCC TGG CA-3′
IL-1β	NM_008361	Forward Reverse	5′-GAA ATG CCA CCT TTT GAC AGT-3′ 5′-GAA GGT CCA CGG GAA AGA CA-3′
IL-13	NM_008355	Forward Reverse	5′-CTG TGT AGC CCT GGA TTC CC-3′ 5′-AGG CCA TGC AAT ATC CTC TGG-3′
IL-33	NM_001164724	Forward Reverse	5′-TGG TCC CGC CTT GCA AAA TA-3′ 5′-GAC GCA GCA AAT GCT TGG AT-3′
TSLP	NM_021367	Forward Reverse	5′-ACT GCA ACT TCA CGT CAA TTA CG-3′ 5′-TTG CTC GAA CTT AGC CCC TTT-3′
GCLC	NM_010295	Forward Reverse	5′-CTA CCA CGC AGT CAA GGA CC-3′ 5′-CCT TCC GGC GTT TCC TCA TA-3′
GST	NM_013541	Forward Reverse	5′-CGG CAA ATA TGT CAC CCT C-3′ 5′-CCT TCC GGC GTT TCC TCA TA-3′
TXN1	NM_011660	Forward Reverse	5′-GCT TGT CGT GGT GGA CTT CT-3′ 5′-AAC TCC CCC ACC TTT TGA CC-3′
GAPDH	NM_001289726	Forward Reverse	5′-GGA GAG TGT TTC CTC GTC CC-3′ 5′-ACT GTG CCG TTG AAT TTG CC-3′
